# Test-Retest Reliability of a Serious Game for Delirium Screening in the Emergency Department

**DOI:** 10.3389/fnagi.2016.00258

**Published:** 2016-11-07

**Authors:** Tiffany Tong, Mark Chignell, Mary C. Tierney, Jacques S. Lee

**Affiliations:** ^1^Interactive Media Lab, Department of Mechanical and Industrial Engineering, University of Toronto, TorontoON, Canada; ^2^Knowledge Media Design Institute, Faculty of Information, University of Toronto, TorontoON, Canada; ^3^Department of Family and Community Medicine, University of Toronto, TorontoON, Canada; ^4^Primary Care Research Unit, Sunnybrook Health Sciences Centre, TorontoON, Canada; ^5^Clinical Epidemiology Unit, Department of Emergency Services, Sunnybrook Health Sciences Center, TorontoON, Canada

**Keywords:** cognitive screening, human factors, serious games, delirium, gerontology, test-retest reliability

## Abstract

**Introduction:** Cognitive screening in settings such as emergency departments (ED) is frequently carried out using paper-and-pencil tests that require administration by trained staff. These assessments often compete with other clinical duties and thus may not be routinely administered in these busy settings. Literature has shown that the presence of cognitive impairments such as dementia and delirium are often missed in older ED patients. Failure to recognize delirium can have devastating consequences including increased mortality (Kakuma et al., 2003). Given the demands on emergency staff, an automated cognitive test to screen for delirium onset could be a valuable tool to support delirium prevention and management. In earlier research we examined the concurrent validity of a serious game, and carried out an initial assessment of its potential as a delirium screening tool (Tong et al., 2016). In this paper, we examine the test-retest reliability of the game, as it is an important criterion in a cognitive test for detecting risk of delirium onset.

**Objective:** To demonstrate the test-retest reliability of the screening tool over time in a clinical sample of older emergency patients. A secondary objective is to assess whether there are practice effects that might make game performance unstable over repeated presentations.

**Materials and Methods:** Adults over the age of 70 were recruited from a hospital ED. Each patient played our serious game in an initial session soon after they arrived in the ED, and in follow up sessions conducted at 8-h intervals (for each participant there were up to five follow up sessions, depending on how long the person stayed in the ED).

**Results:** A total of 114 adults (61 females, 53 males) between the ages of 70 and 104 years (*M* = 81 years, *SD* = 7) participated in our study after screening out delirious patients. We observed a test-retest reliability of the serious game (as assessed by correlation *r*-values) between 0.5 and 0.8 across adjacent sessions.

**Conclusion:** The game-based assessment for cognitive screening has relatively strong test-retest reliability and little evidence of practice effects among elderly emergency patients, and may be a useful supplement to existing cognitive assessment methods.

## Introduction

In many countries, aging populations place serious demands on healthcare systems. The cost of healthcare spending per adult aged 65 years and older has been estimated to be three to five times more than the corresponding cost for younger individuals (Glass and Balfour, 2003; Centers for Disease Control and Prevention, 2013). The rise in healthcare expenditures due to age-related conditions has prompted research on how to minimize costs while maximizing care for adults through early screening, monitoring and intervention methods (Zaslavsky et al., 2012). Cognitive screening of older people is important to monitor chronic (e.g., dementia) or acute (e.g., delirium) changes in cognitive status. In the chronic context, cognitive assessment is needed to monitor risk of dementia for people likely to have cognitive impairment (Petersen et al., 2001). In both primary care and the ED, recognition of cognitive impairment has repeatedly been shown to be poor. In primary care, (Tierney and Lermer, 2010): “the available data indicate that the substantial rates of under-utilization of existing cognitive tools in primary care are mainly due to lack of time, lack of training, lack of tools perceived as helpful, and lack of confidence.”

Cognitive screening is particularly important in the case of elderly patients at risk for delirium. Delirium is a serious, and potentially fatal problem affecting up to 50% of hospitalized seniors, and costing over $164 billion per year in the US as of 2011 (Inouye et al., 2014). Failure to detect delirium is associated with poorer outcomes, including a two-to-three fold increase in mortality (Kakuma et al., 2003; Pun and Ely, 2007). However detection can be improved by routine cognitive testing (Meagher et al., 2001). Patients with intensive care unit delirium have more than a threefold-increased risk of 6-month mortality compared to those without delirium.

Since one of the key properties of delirium is a fluctuating course (Inouye, 1990; Meagher et al., 2001), where a patient may appear normal at one point, and show signs of confusion hours later, repeated cognitive assessment is needed to assess both the onset, and risk of onset, of delirium.

Risk of delirium is also elevated for elderly patients undergoing surgery. Rudra et al. (2006) argued that “good preoperative evaluation should include a formal cognitive assessment in patients at risk of developing delirium.” In long-term care, prevalence of delirium has been reported to range between 1.4 and 70%, depending on diagnostic criteria and on the prevalence of dementia (de Lange et al., 2013). Risk of delirium is particularly high for people with dementia, who are over the age of 85, or who are living in a care facility (de Lange et al., 2013). Delirium has been estimated to be present in 7 to 10% of older patients in the ED (Hustey et al., 2000; Hustey and Meldon, 2002; LaMantia et al., 2014). However, emergency providers identify delirious patients in only 16 to 35% of cases (Hustey et al., 2000). Thus there is a clear need for better screening of delirium in the ED, not only when elderly patients are admitted, but also during their sometimes lengthy stays in the ED where they may transition to a delirious state.

Earlier, we developed a tablet-based serious game for cognitive assessment (Tong and Chignell, 2014). Tong et al. (2016) carried out an initial concurrent validation (with existing methods of clinical assessment) of the game in an ED, finding significant correlations of game performance with scores on the MMSE (Folstein et al., 1975) and the MoCA (Nasreddine et al., 2005). Tong et al. (2016) also found evidence that the serious game may be useful as an initial screen for delirium, with game RT differing significantly between CAM (Inouye et al., 1990) positive and CAM negative patients in a sample of elderly emergency patients. In this paper we examine the test-retest reliability (e.g., Anastasi, 1988) of our serious game as a further investigation of its psychometric properties, with the motivating application being screening for delirium in an ED.

## Background Information

### Cognitive Assessment

The most frequently used clinical tests of cognitive ability in the elderly, such as the MMSE and MoCA, are pencil-and-paper based. The MMSE and MoCA have high test-retest reliabilities, reported between 0.80 and 0.95 for the MMSE (Tombaugh, 2005), and 0.92 for the MoCA (Nasreddine et al., 2005). These assessments are administered by having trained personnel ask patients for verbal or written responses. Thus, they are not designed or suitable for self-assessment in nonclinical environments. They can also be time consuming to carry out in a busy clinical setting such as an ED.

Challenges associated with current methods of cognitive assessment include limited alternate versions of paper-based tests, which can subsequently lead to practice effects. Practice effects have been shown with the MMSE in both healthy adults and those diagnosed with dementia at short test-retest intervals ranging from 10 min to 1.5 weeks (Galasko et al., 1993; Jacqmin-Gadda et al., 1997), and longer intervals of three months (Helkala et al., 2002). Moreover, the MoCA has also demonstrated practice effects in longitudinal performance of healthy older adults (Cooley et al., 2015). The presence of practice effects in cognitive assessments is potentially due to a limited range of questions, and these effects are most prominent on questions evaluating the domains of visual memory, attention, working memory, processing speed, and executive functioning (Cooley et al., 2015). A meta-analysis by Calamia et al. (2012) revealed that practice effects are more pronounced with short test-retest intervals.

Some existing cognitive screening tools have been modified to increase their accessibility and use for different types of patients. For example, the MoCA has an alternative-scoring schema for patients with only a high-school level education (Nasreddine et al., 2005), and the CAM has also been adapted for use in intensive care units (Ely et al., 2001). However, tests tend to be limited in terms of when and how they can be used. For instance, paper-and-pencil tests that require written input (such as the clock drawing task component on the MoCA) may be difficult to complete for patients with limited ranges of motion or other physical disabilities, or for the bed bound.

### Electronic Cognitive Assessment

With the availability of the Internet, and electronic devices such as tablets and smartphones, there has been a shift towards designing electronic cognitive assessments, and translating existing screening tools into a digital medium. The use of technology can provide many benefits, including the ability to record information such as RT and accuracy with precision (Collerton et al., 2007; Wild et al., 2008). Data collection using technology can assist in reducing errors in transcribing paper-based results into digital formats. Once collected, electronic cognitive assessments can also be easily shared between healthcare professionals and patients.

Existing computer-based software for cognitive assessment includes the CANTAB (Cambridge Cognition Ltd, 2014), CNS Vital Signs (Gualtieri and Johnson, 2006), and CAMCI (Saxton et al., 2009). Aside from computer-based testing, other form factors such as smartphones and touch-based tablets have been explored in tools such as the CADi, which screens for dementia (Onoda et al., 2013), the CST, which screens for general cognitive function (Brouillette et al., 2013), and DETECT, which screens for MCI in elderly patients in primary care settings (Wright et al., 2010). The National Institutes of Health (2012) has introduced the NIH Toolbox, which contains an array of tests that monitor neurological and behavioral function (Gershon et al., 2013). The NIH Toolbox requires a test administrator to run and score the tests, which cannot be self-administered by patients.

### Serious Games for Cognitive Assessment

There has been growing interest in the use of serious games to assess cognitive status. Serious games are games designed with a primary purpose other than entertainment (Charsky, 2010). Examples include the ElderGAMES Project (Gamberini et al., 2006), which uses a tabletop setup designed for use by multiple users. Other work by Anguera et al. (2013) has explored the use of driving simulation as a means for brain training and assessment. The use of virtual reality devices has also been explored as a method to assess cognitive function with serious games as exemplified by the work of (Zuchella et al., 2014). Current game-based approaches to cognitive screening are limited by lack of validation with clinical populations and insufficient reliability testing.

In the remainder of this paper we report on a study that assessed the test-retest reliability of a serious game for cognitive assessment, within an ED. One benchmark for comparing the test-retest reliability of the serious game is the test-retest reliability results for the MMSE. Test-retest reliability assessments for the MMSE typically used cognitively intact individuals and test-retest intervals of less than 6 months. The reliability estimates generally fell between 0.80 and 0.95 (Tombaugh and McIntyre, 1992). Tombaugh (2005) examined test-retest reliabilities of the MMSE for people without cognitive impairment over different pairs of four time periods that varied between 1 and 5 years apart. The test-retest reliabilities over these longer time periods were lower, varying between 0.48 and 0.65.

## Materials and Methods

### Procedure

We conducted an observational cohort study between January, 2015 and October, 2015. Our protocol for the study was approved by institutional review boards at the Sunnybrook Health Sciences Centre and the University of Toronto (protocols 070-2013 and 28953, respectively). The study was carried out in accordance with the recommendations of both review ethics boards with written informed consent obtained from all participants.

Trained clinical RAs administered the following standard cognitive assessments during the initial enrolment: MMSE, MoCA, DI (McCusker et al., 2004), RASS (Sessler et al., 2002), a DVT (Kelland, 1996), and a CRT task. Following this, the RA asked patients to play the serious game-based assessment.

During follow up sessions, RAs administered the MMSE, DI, CAM, DVT, and the serious game.

The serious game was a tablet-based version of a go, no-go discrimination task (Yechiam et al., 2006) in the form of a whack-a-mole game (Tong and Chignell, 2014), and there were two primary performance measures: RT and target offset. RT was measured as the time between the appearance of a target and user’s response, and target offset was measured as the pixel distance between the center of the target and the center of the user’s touch.

#### Patient Selection

Potential subjects were screened using the ED Information System at Sunnybrook Hospital part of the Sunnybrook Health Sciences Centre in Ontario, Canada. We approached patients who presented during study hours, regardless of their presenting state. The inclusion criteria for potential subjects included (1) being 70 years of age or older, and (2) present in the ED for a minimum of 8-h.

The exclusion criteria for subjects included: (1) being critically ill (defined by a Canadian Triage Acuity Scale score of 1, (2) having acute pain (a Numeric Rating Scale ≥ 2/10), (3) currently receiving psychoactive medications, (4) having a psychiatric primary presenting complaint, (5) having been previously enrolled, or (6) not speaking English or being unable to follow commands or communicate verbally and (7) having hand injuries preventing use of the tablets. We also screened patients with the CAM and removed patients from the study if they were found to be CAM positive in the initial session, or in any of the follow up sessions. Since patients with delirium will typically have fluctuating cognitive status, they were not considered in assessing test-retest reliability.

### Test-Retest Reliability

The test-retest reliability of the serious game was assessed by conducting follow-ups at regular time intervals a minimum of 8-h apart, on CAM negative patients. Patients varied in the number of follow-ups they participated in, depending on their total time in the ED. During each follow up, the RA administered the MMSE, DI, CAM, and DVT, and asked the patient to play the serious game. For each patient, there was a maximum of five follow-up sessions, in addition to the initial enrolment into the study. Reliability of game scores between pairs of sessions was tested using both Pearson and Spearman correlations.

### Statistical Analysis

The assumption of normality was checked for the MMSE scores, as well as for the serious game median RT and target offset data. Data normality was visually inspected using histograms, P-P and Q-Q plots. Due to the large sample size, tests such as the Kolmogorov-Smirnov and Shapiro-Wilk were not carried out as they are overly sensitive with large sample sizes (Field, 2013). Results from the MMSE were treated as interval data. The median RT data for the serious game was positively skewed and no data transformations were performed. However, the target offset data were normally distributed. Median RTs were used to summarize the RT data in order to reduce the impact of positive skew and outliers on analyses with the RT data. In addition, non-parametric tests were also used as an alternative interpretation of the data without making normality assumptions (Spearman’s *rho*, and the Wilcoxon signed-rank test).

## Results

### Study Sample

A total of 114 patients participated in the study, between the ages of 70 and 104 years (*SD* = 7). There were 61 females, and 53 males in the sample. The average length of stay in the ED was 16.3 h (*SD* = 9.0) (**Table 1**).

**Table 1:** Demographics of the study sample.

Baseline features	
Mean age (years) (*SD)*	81.1 (7.0)
Female (*n*)	61
Male (*n*)	53
Mean length of stay in the ED (hours) (*SD*)	16.3 (9.0)

### Completion Rate

Of the 114 participants who played the serious game in the initial session, 47, 23, and 16 patients (who were assessed as CAM negative played the game in follow up sessions 1, 2, and 3, respectively. Of this set, the numbers of people also completing other assessments, during the initial session and the follow up sessions, is shown in **Table 2**.

**Table 2:** Distribution of cognitive assessment scores and game performance.

Assessment	Initial enrolment	Follow up 1	Follow up 2	Follow up 3	Follow up 4	Follow up 5
MMSE	12 – 30 (*n* = 113)	29 (*n* = 1)	20 (*n* = 1)			
MoCA	10–30 (*n* = 14)					
RASS	–1 to 1 (*n* = 114)					
DI	0–7 (*n* = 113)	0–3 (*n* = 9)	0–4 (*n* = 5)	0 (*n* = 2)	0–2 (*n* = 2)	2 (*n* = 1)
DVT	81–103 (*n* = 27)	86–101 (*n* = 12)	85–103 (*n* = 6)	92–101 (*n* = 3)	95–100 (*n* = 3)	
CRT RT (sec)	0.78–11.68 s (*n* = 93)					
CRT Acc (%)	40–95 (*n* = 93)					
Serious Game Median RT (s)	0.62–4.50 (*n* = 114)	0.63–1.91 (*n* = 47)	0.59–1.10 (*n* = 23)	0.50–1.37 (*n* = 16)	0.76–0.86 (*n* = 2)	0.82 (*n* = 1)
Serious Game Target Offset (px)	243.50–449.00 (*n* = 114)	218.00–409.00 (*n* = 47)	202.00–365.50 (*n* = 23)	162.00–339.00 (*n* = 16)	306.50–331.00 (*n* = 2)	306.00 (*n* = 1)

### Cognitive Assessment Demographics

At initial enrolment, MMSE scores ranged from 12 to 30, and MoCA scores ranged from 10 to 30. The ranges and distribution of scores for each cognitive assessment based on each session are displayed in **Table 2**. The MoCA, RASS, and CRT were only carried out during initial enrolment and thus their test-retest reliability was not assessed. Some MMSE assessment and game performance data was missing for participants who either refused to complete the assessment, or were fatigued or sleeping when RAs came to assess them.

### Test-Retest Reliability

**FIGURE 1:**
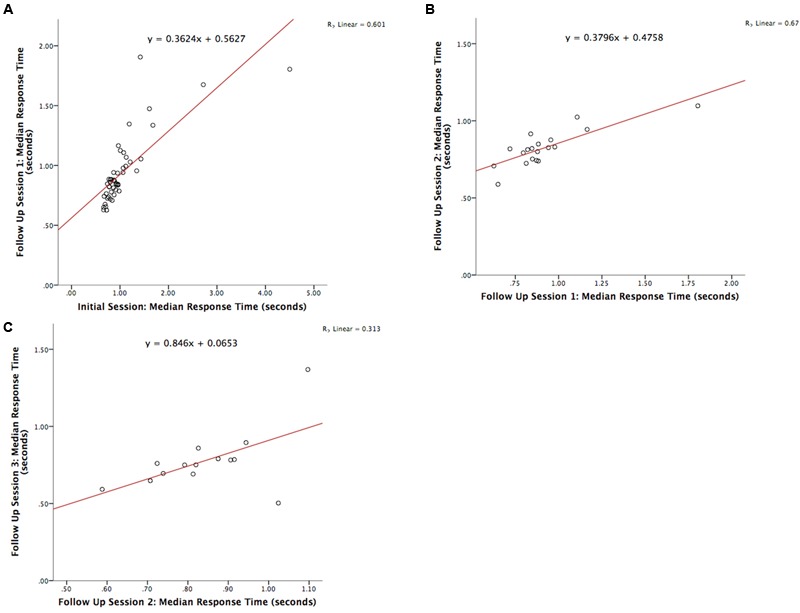
**Graph depicting the serious game median RT between: **(A)** initial enrolment compared to follow up session 1 (*r* = 0.776, *p* < 0.001), **(B)** follow up session 1 compared with 2 (*r* = 0.821, *p* < 0.001), and **(C)** follow up session 2 compared with 3 (*r* = 0.560, *p* = 0.037)**.

**FIGURE 2:**
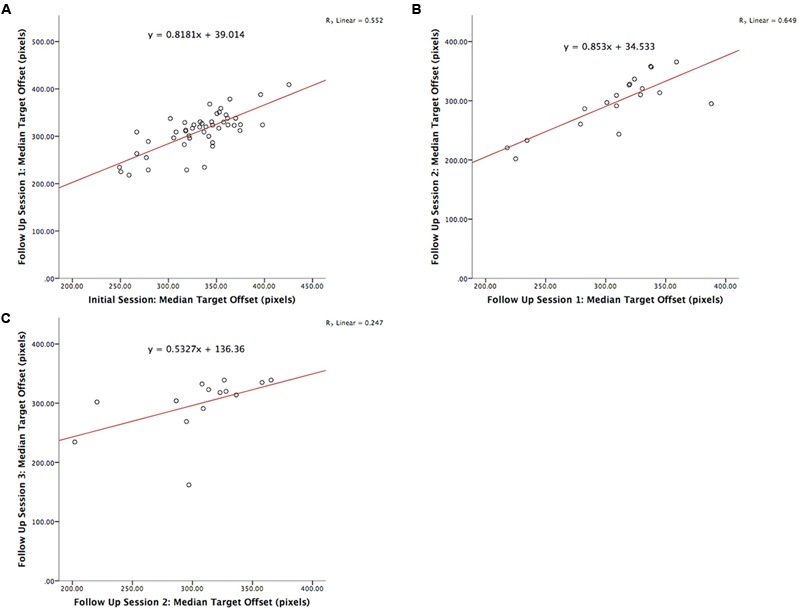
**Graph depicting the serious game median target offset between: **(A)** initial enrolment compared to follow up session 1 (*r* = 0.742, *p* < 0.001), **(B)** follow up session 1 compared 2 (*r* = 0.806, *p* < 0.001), and **(C)** follow up session 2 compared 3 (*r* = 0.497, *p* = 0.071)**.

The test-retest reliability of the serious game was investigated by calculating two-tailed Pearson’s *r* correlations between pairs of sessions (**Table 3** shows correlations for median RT and **Table 4** shows correlations for median target offsets). While **Table 3** shows all possible pairwise correlations, the three correlations between adjacent time periods are shaded and each correlation in these shaded time periods was significant (*p* < 0.05) with *r*-values ranging between 0.56 and 0.82. Corresponding scatterplots for the three adjacent pairs of sessions are shown in **Figure 1**. A similar correlation analysis was carried out using the serious game median target offset values (**Table 4**), with corresponding scatterplots between adjacent time periods being shown in **Figure 2**. All adjacent pairs of sessions had strong correlations, with *r*-values varying between 0.49 and 0.80.

**Table 3:** Relationships between sessions on serious game median RT, was determined using two-tailed Pearson’s *r* correlations.

	Initial enrolment	Follow up 1	Follow up 2	Follow up 3
Initial enrolment	1	0.776^∗∗^ *p* < 0.001 *n* = 47	0.594^∗∗^ *p* = 0.003 *n* = 23	0.862^∗∗^ *p* < 0.001 *n* = 15
Follow up 1		1	0.821^∗∗^ *p* < 0.001 *n* = 19	0.821^∗∗^ *p* = 0.001 *n* = 13
Follow up 2			1	0.560^∗^ *p* = 0.037 *n* = 14
Follow up 3				1

*Shaded gray areas highlight adjacent sessions. ^∗^*p* < 0.05, ^∗∗^*p* < 0.01.*

**Table 4:** Relationship between serious game median target offset between each determined using two-tailed Pearson’s *r* correlations.

	Initial enrolment	Follow up 1	Follow up 2	Follow up 3
Initial enrolment	1	0.742^∗∗^ *p* < 0.001 *n* = 47	0.658^∗∗^ *p* = 0.001 *n* = 23	0.265 *p* = 0.340 *n* = 15
Follow up 1		1	0.806^∗∗^ *p* < 0.001 *n* = 19	0.325 *p* = 0.279 *n* = 13
Follow up 2			1	0.497 *p* = 0.071 *n* = 14
Follow up 3				1

*Shaded gray areas highlight adjacent sessions. ^∗^*p* < 0.05, ^∗∗^*p* < 0.01.*

The test-retest reliability of the serious game median RT and median target offset were recalculated using Spearman’s *rho* correlations (**Tables 5** and **6**) at each follow up. The *rho*-values were all significant (*p* < 0.05) across the three adjacent follow-up sessions for both RT (*rho*-values ranging between 0.56 and 0.85) and target offset (*rho*-values between 0.68 and 0.77).

**Table 5:** Relationships between sessions on serious game median RT, as determined using two-tailed Spearman’s *rho* correlations.

	Initial enrolment	Follow up 1	Follow up 2	Follow up 3
Initial enrolment	1	0.853^∗∗^ *p* < 0.001 *n* = 47	0.534^∗∗^ *p* = 0.009 *n* = 23	0.618^∗∗^ *p* = 0.014 *n* = 15
Follow up 1		1	0.741^∗∗^ *p* < 0.001 *n* = 19	0.588^∗∗^ *p* = 0.035 *n* = 13
Follow up 2			1	0.560^∗^ *p* = 0.037 *n* = 14
Follow up 3				1

*Shaded gray areas highlight adjacent sessions. ^∗^*p* < 0.05, ^∗∗^*p* < 0.01.*

**Table 6:** Relationship between serious game median target offset, between each determined using two-tailed Spearman’s *rho* correlations.

	Initial enrolment	Follow up 1	Follow up 2	Follow up 3
Initial enrolment	1	0.685^∗∗^ *p* < 0.001 *n* = 47	0.451^∗∗^ *p* = 0.031 *n* = 23	0.340 *p* = 0.216 *n* = 15
Follow up 1		1	0.777^∗∗^ *p* < 0.001 *n* = 19	0.484 *p* = 0.094 *n* = 13
Follow up 2			1	0.741^∗∗^ *p* = 0.002 *n* = 14
Follow up 3				1

*Shaded gray areas highlight adjacent sessions. ^∗^*p* < 0.05, ^∗∗^*p* < 0.01.*

### Practice and Fatigue Effects

**FIGURE 3:**
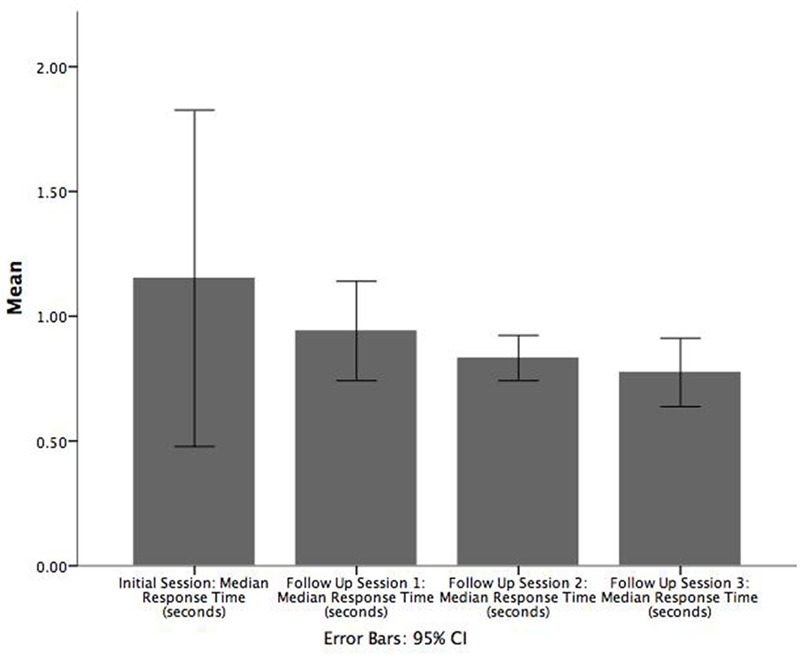
**Bar chart illustrating the mean of median RT (seconds) across each follow up session.** Error bars are 95% CI.

We carried out inferential tests to assess the statistical significance of possible practice/learning effects. Three paired *t*-tests (two-tailed) were carried out to determine if there was a difference in game median RT between (1) initial enrolment and follow up 1, (2) follow up sessions 1 and 2, and (3) follow up sessions 2 and 3. Bar charts corresponding to these comparisons are shown in **Figure 3** where it can be seen that there is a decreasing trend in median RT across the sessions.

There was no significant difference between the initial enrolment and first follow up session or between the second and third follow up sessions. However, patients had a significantly greater game median RT in the first follow up session (*M* = 0.9, *SE* = 0.06) than in the second follow up session (*M* = 0.8, *SE* = 0.03), *t*(18) = 2.384, *p* = 0.028, *r* = 0.821. In contrast, the corresponding Wilcoxon signed-rank tests indicated that all pairs of adjacent sessions were significant: (1) initial enrolment and follow up session 1 (*Z* = -2.374, *p* = 0.018), (2) follow up sessions 1 and 2 (*Z* = -2.696, *p* = 0.007), and (3) follow up sessions 2 and 3 (*Z* = -2.103, *p* = 0.035).

**FIGURE 4:**
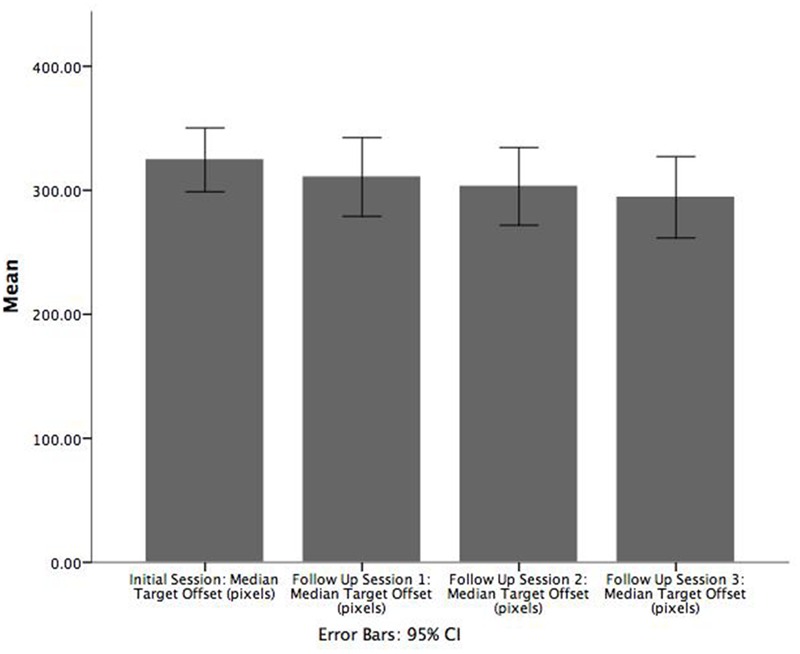
**Bar chart illustrating the mean of median target offset (pixels) between initial enrolment compared to follow up session 1.** Error bars are 95% CI.

Paired samples *t*-tests were also conducted using the game target offset values for patients to examine the difference in performance between adjacent sessions (i.e., between the initial session and follow up 1, between follow up sessions 1 and 2, and between follow up sessions 2 and 3). On average, patients were significantly less accurate in the initial enrolment (*M* = 331.9, *SE* = 5.6) versus follow up session 1 (*M* = 310.5 *SE* = 6.1), *t*(47) = 5.050, *p* = 0.000, *r* = 0.743 (**Figure 4**). However, significant differences in game target offset values were not observed for the other two comparisons. A corresponding Wilcoxon signed-rank test showed that game target offset between initial enrolment and follow up session 1 was significantly different (*Z* = -4.441, *p* < 0.001). Participants had smaller target offset values in the first follow up sessions compared to initial enrolment. As with the *t*-tests, the Wilcoxon signed-rank tests did not show significant differences in target offset between follow up sessions 1 and 2, nor between follow up sessions 2 and 3.

**FIGURE 5:**
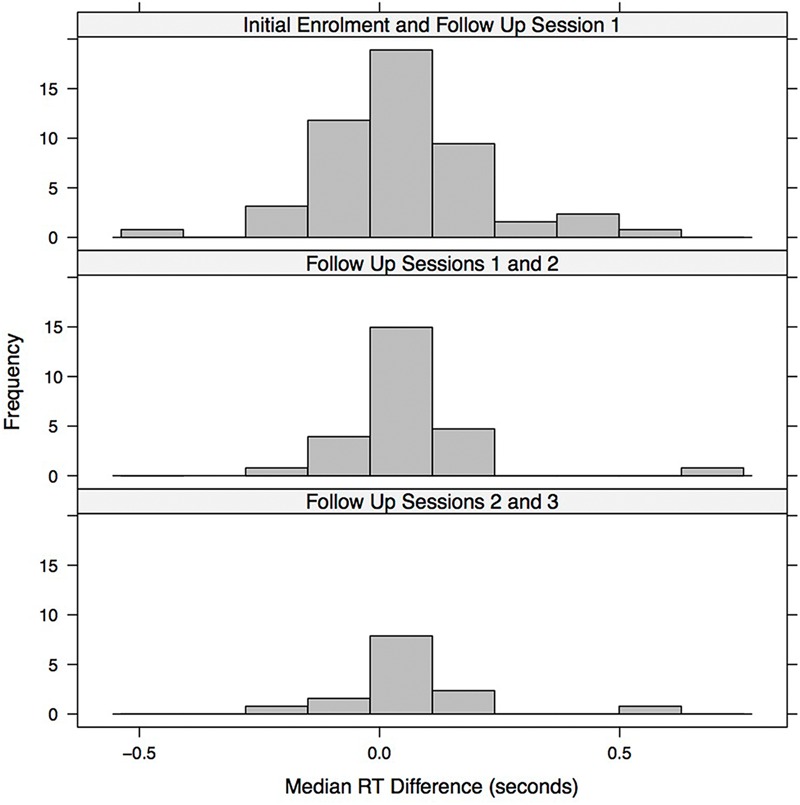
**Histogram illustrating the median RT difference (seconds) between successive session.** Each bin represents 0.05 s.

**Figure 5** visualizes potential practice and fatigue effects across successive follow up sessions. The figure shows histograms of the median RT differences (within participants) between successive sessions. In the initial enrolment and first follow up session, one patient with a difference greater than 1 s (the difference was greater than 2.5 s for this patient, but the 1 s difference was used as the cut-off) was omitted from the histogram. The histograms were then scaled to be on the same x-axis with the same time bin sizes (each time bin had a width of 50 ms) and were lined up vertically to facilitate visual comparison. We assume that patients with a difference in median RT that was greater than zero in the subsequent session were in poorer condition or were experiencing fatigue. In contrast, for patients with a difference less than zero, there was likely a practice effect, as they were speeding up in the subsequent session. Since the distribution of differences in median RT between adjacent sessions (within individuals) is reasonably well balanced around the no difference (0 s) point there is little evidence of a genuine learning effect in game RT performance. Instead, the reduction in game median RT in later sessions (as indicated by significant *t*-tests and Wilcoxon signed-rank tests) is likely due, in most part, to slower participants dropping out of the study either because they were treated more quickly or because they were less willing or able to participate in the later sessions.

## Discussion

The present findings demonstrate the test-retest reliability of our game-based screening tool with an elderly emergency population. We observed strong relationships between all possible pairs of administrations of our serious game (initial enrolment, and follow up sessions 1 through 3) with *r*-values generally ranging between 0.56 and 0.82 for both median RT, and median target offset values (**Tables 3** and **4**). Similar Spearman’s *rho*-values ranges were observed for game median RT, and for median target offset (see **Tables 5** and **6**), respectively.

In the initial enrolment, we observed a wide range of MMSE (12–30) and MoCA (10–30) scores. Patients with possible dementia (MMSE scores below 24) (O’Connor et al., 1989) and MCI (MoCA scores below 23) (Luis et al., 2009) were still able to play our serious game. This suggests that people with cognitive impairments can use our game-based cognitive assessment.

The game median RT performance over sessions, within patients, was relatively consistent with the histograms of within patient RT differences between adjacent sessions (**Figure 5**) balanced around the zero difference value. Thus there is no evidence for the presence of a practice/learning effect.

We expected that many patients who were high performing would be assessed and discharged prior to their second assessment and that game performance would likely get worse in later sessions. However, this was not the case. Thus it appears that the sample of patients who remained in the ED over an extended period of time, remained CAM negative, and who were able to play the game, were comparatively fit. They had better game performance than the patients who dropped out of the study after the earlier sessions. With respect to the fact that within subjects there was no tendency for either speeding up or slowing down across sessions, it is possible that practice/learning effects (speeding up) precisely matched the fatigue effects (slowing down). However, a more parsimonious explanation might be that there were no practice or fatigue effects in this case, which would be a beneficial property of the game if it can be further verified in future research.

For cognitive assessment, the reliability of game RT is of most interest since game RT was found to correlate better (than game target offset) with other clinical assessments in the previous concurrent validity study (Tong et al., 2016). The test-retest reliability correlations obtained with game RT in this study were comparable with the test-retest correlations obtained by Tombaugh (2005) in his study of the test-retest reliability of the MMSE. However, in the present study follow up sessions were separated by 8-h, whereas in Tombaugh’s (2005) study the different time periods were separated by one or more years. In addition, the MoCA has been shown to have a high test-retest reliability of 0.92 over a period of around a month (Nasreddine et al., 2005), which is significantly longer than the 8-h separation between follow ups in the present study. In cases where short test-retest time intervals were used, test-retest reliability of the MMSE was reported to be much higher (between 0.80 and 0.95). There are likely a number of reasons why previous published values may tend to overestimate the value of the test-retest reliability of the MMSE, of which two are considered here. First, the MMSE studies generally selected people who were not assessed to be cognitively impaired (e.g., the participants would have had MMSE scores relatively close to the top end of the MMSE scale). This would have resulted in a compressed scale, making it more likely that scores within individuals would tend not to change (in contrast to game RT where the RTs may vary over many milliseconds). Second, since the MMSE items are identical and since the focus is on unimpaired individuals, scores will tend to stay the same or possibly improve due to practice effects and the ability to remember items on the test. In contrast to the MMSE, a patient cannot remember an exact sequence of events in the serious game that we used, since where and when targets appear was varying, and determined probabilistically. In the present study, we found little evidence of practice effects in game median RT performance, when time differences within individuals were assessed (**Figure 5**).

### Limitations and Future Work

Our study focused on patients admitted to one hospital ED. It is possible that somewhat different findings might have been obtained in a different ED. In future studies, research should explore a more diverse patient population so as to improve the generalizability of the results. While we did not observe a strong learning (practice) effect in game performance between sessions, it is likely that there was an initial learning effect when patients first started using the game. We did not attempt to assess the initial learning effect in this study. Instead we allowed patients to do some initial practice with the game, with feedback and encouragement provided by the RAs, before the patient played the game to make the assessment. In contrast to the apparent lack of practice effects for the serious game median RT, practice effects have been found in “standard” cognitive assessments such as the MoCA and MMSE.

While significant, the test-retest reliabilities observed here for the serious game are lower than the corresponding reliabilities reported for the MMSE (for short time periods of up to a few months). However, the game provides a much wider range of scores, since RT is measured in milliseconds and it is not possible to memorize the answers to questions as it may be for tests such as the MMSE.

We did not assess inter-rater reliability for the CAM (used to screen out delirious patients in this study). However, the CAM has been shown to have a high inter-rater reliability (Bhat and Rockwood, 2007). Moreover, we also observed a high rate of loss-to-follow-up due to carrying out assessments every 8-h. Future studies should consider using shorter latencies between sessions (e.g., every 1-h) in order to improve the chances of detecting delirium.

## Conclusion

We have demonstrated that our game-based screening tool is a reliable tool (in terms of test-retest reliability) for measuring cognitive status, and that it can be used independently by patients in emergency care after a few minutes of training (at most). In related work we have also begun the process of validating our game-based scrreening tool by establishing that game median RT is significant correlated with standard clinical assessments such as the MMSE, and MoCA (Tong et al., 2016). Taken together, the present work and our previous study demonstrate that our serious game for cognitive assessment has good levels of both concurrent validity and test-retest reliability. The game is also usable, and can be self-administered by patients. While the game appears to have lower test-retest reliability than the MMSE, it does not seem to have practice effects if people are given a short amount of initial training, and it provides a wider range of scores and no opportunities for memorizing answers. While the serious game has yet to be fully validated for routine assessment of cognitive decline, this research opens the way to further explore self-administration by patients of a cognitive screening tool that is able to track their progress over time. In busy environments such as the ED, this type of serious game for self-administered cognitive assessment could, in the future, assist both healthcare providers and patients by providing critical information on a patient’s cognitive status over time. The game should be a useful supplement to tests such as the MoCA, and MMSE in situations where it may be difficult or impractical to use those existing assessments.

## Author Contributions

TT designed and developed the game, carried out the statistical analyses, and wrote the first draft of the manuscript. JL designed the clinical study and supervised the data collection. MT assisted with the selection of clinical assessments used in the study and in the interpretation of the results. MC assisted in the research design and statistical analysis. All of the authors participated in the editing and revision of the manuscript.

## Conflict of Interest Statement

The authors declare that the research was conducted in the absence of any commercial or financial relationships that could be construed as a potential conflict of interest.

## Abbreviations

CAM: Confusion Assessment Method
CANTAB: Cambridge Automated Neuropsychological Testing Battery
CRT: Choice Reaction Time
DI: Delirium Index
DVT: Digit Vigilance Task
ED: Emergency Department
M: Mean
MMSE: Mini Mental State Exam
MoCA: Montreal Cognitive Assessment
RA: Research Assistant
RASS: Richmond Agitation-Sedation Scale
RT: Response Time
SD: Standard Deviation
SE: Standard Error
